# Effect of glutaraldehyde based cross-linking on the viscoelasticity of mitral valve basal chordae tendineae

**DOI:** 10.1186/s12938-018-0524-2

**Published:** 2018-07-13

**Authors:** M. Constable, H. E. Burton, B. M. Lawless, V. Gramigna, K. G. Buchan, D. M. Espino

**Affiliations:** 1 Department of Mechanical Engineering, University of Birmingham, Birmingham, B15 2TT UK; 2 PDR, International Centre for Design and Research, Cardiff Metropolitan University, Cardiff, CF5 2YB UK; 3 University of Magna Graecia, Catanzaro, Italy; 4 IBFM, National Research Council, Germaneto, Catanzaro Italy; 5 Department of Cardiothoracic Surgery, Aberdeen Royal Infirmary, Foresterhill, Aberdeen, AB25 2ZN UK

**Keywords:** Chordae tendineae, Dynamic mechanical analysis, Glutaraldehyde, Mechanical properties, Mitral valve, Viscoelasticity

## Abstract

**Background:**

Mitral valve failure can require repair or replacement. Replacement bioprosthetic valves are treated with glutaraldehyde prior to implantation. The aim of this study was to determine the changes in mechanical properties following glutaraldehyde fixation of mitral valve chordae.

**Methods:**

To investigate the impact of glutaraldehyde on mitral valve chordae, 24 basal chordae were dissected from four porcine hearts. Anterior and posterior basal (including strut) chordae were used. All 24 chordae were subjected to a sinusoidally varying load (mean level 2N, dynamic amplitude 2N) over a frequency range of 0.5–10 Hz before and after glutaraldehyde treatment.

**Results:**

The storage and loss modulus of all chordal types decreased following glutaraldehyde fixation. The storage modulus ranged from: 108 to 119 MPa before fixation and 67.3–87.4 MPa following fixation for basal chordae; 52.3–58.4 MPa before fixation and 47.9–53.5 MPa following fixation for strut chordae. Similarly, the loss modulus ranged from: 5.47 to 6.25 MPa before fixation and 3.63–4.94 MPa following fixation for basal chordae; 2.60–2.97 MPa before fixation and 2.31–2.93 MPa following fixation for strut chordae.

**Conclusion:**

The viscoelastic properties of mitral valve chordae are affected by glutaraldehyde fixation; in particular, the reduction in storage moduli decreased with an increase in chordal diameter.

## Background

The mitral valve is an atrioventricular valve located in the left side of the heart [1, 2]. Its function is to enable the unidirectional flow of blood between the left atrium and left ventricle [3–5]. The subvalvular apparatus includes the anterior and posterior leaflets, two papillary muscles and chordae tendineae [4, 6–8]. Chordae connect the papillary muscles to the anterior and posterior leaflets [9]. Chordae can be categorised as marginal or basal. Marginal chordae insert into the free edge of the leaflet and are typically thinner and less extensible than other chordae [6]. Basal chordae are thicker in diameter and insert away from the free edge of the leaflet but between the free edge and the mitral annulus [10]. Strut chordae are thick basal chords which insert into the anterior leaflet only [6, 9]; whereas, commissural chordae branch radially and insert into both leaflets at the commissures between anterior and posterior leaflets [9, 11]. Distinct chordal categories enable specific mechanisms of mitral valve competence to be preserved [12].

The importance of the mechanical and material properties of chordae tendineae relates to its significance in improving the surgical procedure for the correction of mitral valve regurgitation. Chordal failure can lead to the need for surgical correction, of which two approaches are available: surgical repair [10, 13], or surgical replacement. Surgical repair allows for retention of native chordae, providing a less invasive procedure [6, 10, 14–16]. Alternatively, surgical replacement involves replacing the natural valve with an alternative including bioprosthetic or mechanical valves, and homografts [17]. Bioprosthetic valves may be recommended for older patients to avoid the need of anticoagulants associated with mechanical valves [18]. Due to the prevalence of heart failure in the aged population, there is interest in the functionality and durability of bioprosthetic replacement [19].

Clinically, mitral valves are replaced using porcine aortic valves where they are inserted into the mitral valve position. Due to this, studies have predominantly focused on aortic valve replacements where tissues are treated with glutaraldehyde as a fixative. Glutaraldehyde is used to prevent tissue degradation, reduce antigenicity, and improve durability [17, 20–22]. However, such treatments are associated with structural alterations, notably an increase in tissue stiffness [20, 22]. The effects of glutaraldehyde treatment on biological tissues are the focus of several studies due to its use in bioprostheses. However, studies investigating the effects of glutaraldehyde treatment on porcine aortic valves have led to seemingly contradictory findings. For instance, under tension, glutaraldehyde has little impact on tissue stiffness [23]. Conversely, under bending and shear tissue stiffness has been seen to increase [22, 24]. Uniaxial testing also highlighted a decrease in tissue extensibility following glutaraldehyde fixation [25]. Though aortic valves are commonly used for mitral valve replacement, studies investigating the use of bioprosthetic alternatives have concluded positive results; for instance, the use of bovine pericardium as stentless mitral valve replacements [26].

Understanding the effects of glutaraldehyde on the mechanical properties of chordae is crucial in determining the feasibility of using, say, porcine mitral valves for bioprosthetics. However, studies evaluating the effects of glutaraldehyde treatment of mitral valve chordae have been more limited, with one study finding that glutaraldehyde treatment reduced the ultimate tensile strength (UTS) of marginal chordae [17]. Another study demonstrated that glutaraldehyde fixation only affected a portion of the non-linear stress–strain relationship of chordae [27]. Further, chordae are viscoelastic and exposed to dynamic loading conditions [11, 28, 29] yet the viscoelastic properties of glutaraldehyde treated mitral valve chordae are unknown.

A viscoelastic material can be characterised by its storage and loss modulus [11, 30, 31]. The elastic properties of the material are characterised by its ability to store energy, known as the storage modulus. The loss modulus of a material characterises the ability of the material to dissipate energy. Dynamic Mechanical Analysis (DMA) is a technique which enables the characterisation of viscoelastic materials, in terms of storage and loss moduli, under dynamic loading conditions [32–35].

The aim of this study is to assess the effects of glutaraldehyde fixation on the viscoelastic properties of mitral valve chordae tendineae. The viscoelastic properties of chordae have been characterised using DMA at frequencies relevant to physiological and patho-physiological heart rates [11]. For this study, porcine basal chordae have been assessed. However, given the potential for regional variation in mitral valve mechanical properties [36], anterior and posterior basal chordae have been compared, as well as basal and strut chordae, in terms of their dynamic viscoelasticity.

## Methods

### Tissue preparation

**Fig. 1:**
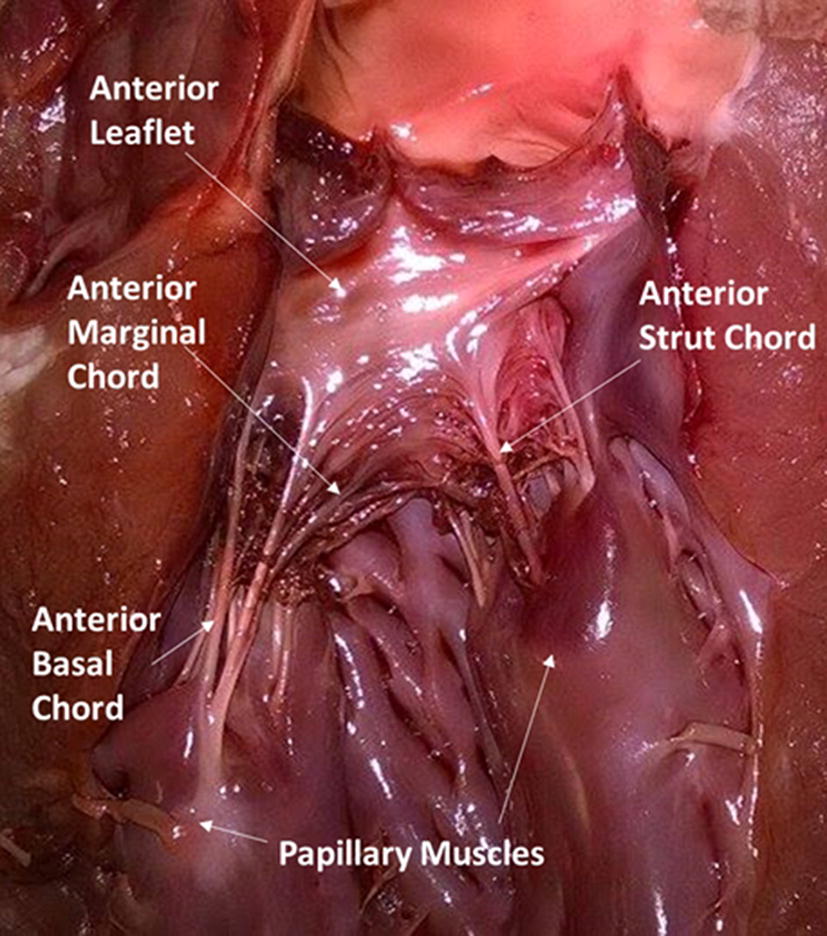
Porcine specimen. The different chordal types have been identified on the anterior mitral valve leaflet

Chordae were obtained from four porcine hearts. Hearts were frozen upon extraction and delivered frozen and sealed by Fresh Tissue Supplies (Fresh Tissue Supplies, East Sussex, UK); no animals were sacrificed specifically for this study. They were stored at – 40 °C wrapped in tissue paper coated in Ringer’s solution following established procedures [11, 37]. Freezing biological tissues using this method does not adversely affect their mechanical properties [38, 39]. Six chordae (2 strut, 2 anterior basal, and 2 posterior basal) from each heart were obtained for testing (Fig. 1).

### DMA

Twenty-four chordae were subjected to DMA using a Bose ElectroForce 3200 testing machine (Bose Corporation, Electroforce Systems Group, Minnesota, USA) previously used for mechanical testing of mitral valve chordae [11, 37] and leaflets [36]. Length and chordal diameter were measured using ImageJ software (ImageJ 1.0, Maryland, USA); images were acquired of chordae when placed within the grips of the testing machine, from which measurements were taken using ImageJ. Chordae were gripped by using emery paper (P400 with two smaller rectangles of P240) to coat the grips of the testing machine, similar to that used previously [11, 33, 37].

Chordae viscoelastic properties are frequency dependent [11], they were sinusoidally loaded between 1 and 3N over a frequency sweep. Specimens were preloaded sinusoidally at 1 Hz for 100 cycles. Subsequently, a frequency sweep of 0.5, 1, 1.2, 3.5, 5, 7, and 10 Hz was used for testing. Chordae were tested at 1.2 Hz to correspond to the 70 beats/min used to represent in vivo conditions, whilst 3.5 and 7 Hz were estimated to match maximum in vivo strain rates [11, 40, 41]. Mitral valve components have been characterised up to 10 Hz of loading [36], hence, the highest loading frequency. Specimens were tested under the same loading protocols, and frequency sweep, before and after glutaraldehyde treatment (“Fixation of chordae” section). Specimens were maintained hydrated throughout testing using Ringer’s solution [11, 33, 37].

At each frequency of loading, an oscillating force (1–3 N) was applied and the out-of-phase displacement was measured and recorded using WinTest 4.1 DMA software (Bose Corporation, Electroforce Systems Group, Minnesota, USA). The WinTest DMA software conducted a Fast Fourier Transform (FFT) of the force and displacement waves at each frequency. The magnitude of the load (F*), displacement (d*), and the phase angle (*δ*) were then determined [31]. Subsequently, the complex stiffness is calculated using Eq. (1) and storage and loss stiffness are derived (Eqs. 2 and 3, respectively).

1 $${k^*} = \frac{F^*}{d^*}$$

2 $$k' = {k^*}\cos \delta \;$$

3 $$k'' = {k^*}\sin \delta$$

where $k'$ and $k''$ refer to the storage and loss stiffness respectively, and k^*^ is the magnitude of the complex stiffness [11, 31]. The storage and loss moduli are then calculated by normalising for a given shape, using a shape factor (*S*); Eqs. 4 and 5, respectively.

4 $$E' = \frac{{{k^*}\cos \delta }}{S}\;$$

5 $$E'' = \frac{{{k^*}\sin \delta }}{S}\;$$

where $E'$ and $E''$ are the storage and loss moduli, respectively. Chordae have been approximated to be cylindrical [11, 37], and the shape factor was defined by:

6 $$S = \frac{{\pi {d^2}}}{4l}\;$$

where *d* is the average diameter of the chord and *l* is its length [11, 37].

### Fixation of chordae

Chordae were fixed using 0.6% glutaraldehyde (Fluka Analytical, Sigma Aldrich, St Louis, MO, USA) solution diluted with 0.2 M PBS (Phosphate Buffered Solution; Sigma Aldrich, Darmstadt, Germany); as previously used with biological tissues [22, 27, 42]. Bioprosthetic replacements are typically fixed at 0.6% glutaraldehyde concentration [17, 25, 27]. Samples were submerged within 5 ml of the glutaraldehyde solution for 1 h as recommended elsewhere [43–45]. Following fixation, specimens were washed thrice in 0.2 M PBS for 10 min to remove excess glutaraldehyde [46].

### Data analysis

Statistical analysis was performed using SigmaPlot v12.0 (SYSTAT, San Jose, CA, USA). Linear regression analysis was used to determine the frequency dependent nature of the storage and loss moduli of each chord (*p* < 0.05). Significant differences (*p* < 0.05) in the storage and loss moduli pre-and post-fixation were determined using the Wilcoxon Signed Rank test for paired non-parametric data. Non-parametric tests were used due to the small sample size (four hearts; i.e. four independent measurements).

A Kruskal–Wallis one-way analysis of variance (ANOVA) on ranks was used to determine any differences between basal chordal types (i.e. anterior, posterior and strut chordae). Significant differences (*p* < 0.05) were evaluated using Tukey’s multiple comparisons test.

## Results

### Storage modulus

The storage modulus for all specimens was frequency dependent, where the storage modulus increased with increasing frequency. Regression analysis showed that the storage modulus, $E'$, was proportional to the natural logarithm of frequency, *f* (Eq. 7). The storage modulus is represented by the function below where *A* and *B* are coefficients (Table 1).

**Table 1:** Linear regression results for the storage and loss modulus of normal chordae

Specimen	Storage modulus				Loss modulus			
	*E′ *= *Aln(f) *+ *B*				*E″ *= *Cln(f) *+ *D*			
	A	B	r²	p value	C	D	r²	p value
Anterior strut 1-1	3.101	69.084	0.998	< 0.001	0.0188	0.588	0.332	0.175
Anterior strut 1-2	4.781	96.175	0.997	< 0.001	0.0313	0.821	0.769	0.01
Anterior strut 2-1	1.404	47.327	0.997	< 0.001	0.176	3.891	0.878	0.002
Anterior strut 2-2	1.179	38.102	0.983	< 0.001	0.148	3.074	0.888	0.001
Anterior strut 3-1	1.598	57.632	0.981	< 0.001	0.124	3.906	0.746	0.012
Anterior strut 3-2	1.482	47.762	0.992	< 0.001	0.153	3.358	0.84	0.004
Anterior strut 4-1	1.484	40.925	0.986	< 0.001	0.0954	3.406	0.912	< 0.001
Anterior strut 4-2	1.198	32.502	0.988	< 0.001	0.105	2.792	0.921	< 0.001
Anterior basal 1-1	3.71	111.136	0.99	< 0.001	0.00947	0.829	0.112	0.463
Anterior basal 1-2	6.435	165.142	0.999	< 0.001	− 0.108	1.545	0.932	< 0.001
Anterior basal 2-1	0.589	31.84	0.935	< 0.001	0.136	2.415	0.897	0.001
Anterior basal 2-2	3.808	114.516	0.992	< 0.001	0.239	8.209	0.727	0.015
Anterior basal 3-1	5.424	168.826	0.986	< 0.001	0.231	12.597	0.521	0.067
Anterior basal 3-2	1.843	99.125	0.993	< 0.001	− 0.00601	7.375	0.00499	0.88
Anterior basal 4-1	2.449	68.879	0.998	< 0.001	0.211	5.996	0.837	0.004
Anterior basal 4-2	3.338	121.975	0.996	< 0.001	0.0738	8.724	0.432	0.108
Posterior basal 1-1	7.593	164.053	0.996	< 0.001	− 0.022	1.123	0.45	0.099
Posterior basal 1-2	5.322	148.988	0.994	< 0.001	− 0.022	0.979	0.434	0.108
Posterior basal 2-1	1.719	68.808	0.989	< 0.001	0.22	5.155	0.838	0.004
Posterior basal 2-2	3.278	88.362	0.996	< 0.001	0.188	7.497	0.813	0.006
Posterior basal 3-1	2.808	106.084	0.986	< 0.001	0.121	7.862	0.47	0.089
Posterior basal 3-2	2.135	88.329	0.997	< 0.001	0.0105	6.01	0.0533	0.618
Posterior basal 4-1	2.87	97.393	0.987	< 0.001	− 0.107	7.42	0.762	0.01
Posterior basal 4-2	3.489	125.307	0.998	< 0.001	0.0419	8.824	0.105	0.478

Results are statistically significant if *p* < 0.05

7 $$E' = A\ln \left( f \right) + B\;$$

**Fig. 2:**
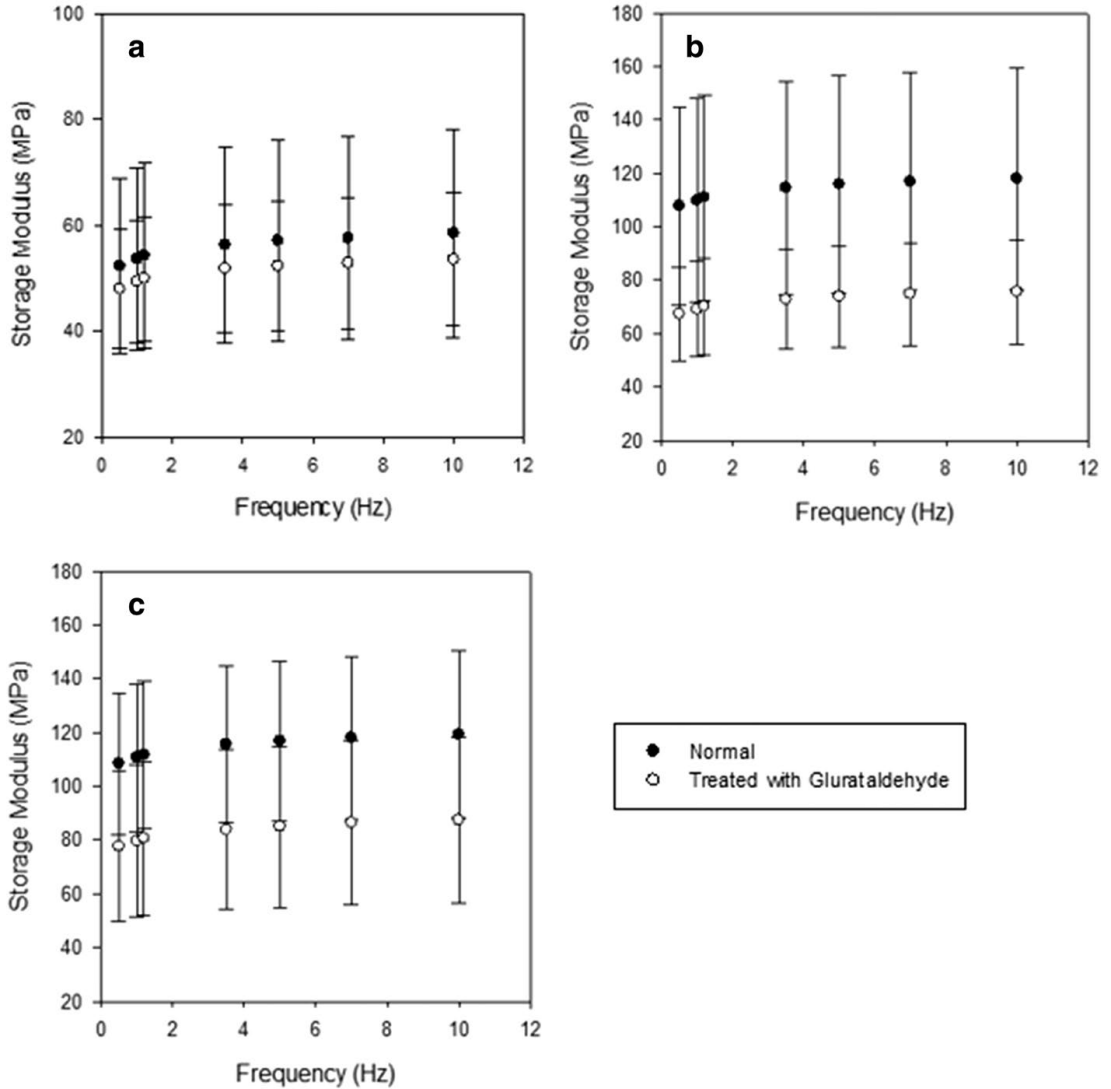
The effects of glutaraldehyde fixation on the storage modulus of **a** Anterior strut chordae, **b** Anterior basal chordae, and **c** Posterior basal chordae. Points represent the sample means with 95% confidence intervals included. Means were calculated based on 8 samples from 4 different hearts for each chordal type. Black dots refer to unfixed chordae, while white dots represent glutaraldehyde treated chordae

The mean storage modulus increased: from 52.3 MPa at 0.5 Hz to 58.4 MPa at 10 Hz (Fig. 2a) for anterior strut chordae; from 108 MPa at 0.5 Hz to 118 MPa at 10 Hz for anterior basal chordae (Fig. 2b); from 109 MPa at 0.5 Hz to 119 MPa at 10 Hz for posterior basal chordae (Fig. 2c).

Fixation decreased the storage modulus for all chordal types across the frequency range (Tables 2 and 3), but storage moduli still increased with frequency. Following glutaraldehyde fixation, the mean storage modulus for anterior strut chordae increased from 47.9 MPa at 0.5 Hz to 53.5 MPa at 10 Hz. The mean storage modulus of fixed anterior and posterior basal chordae increased from 67.3 MPa and 77.7 MPa at 0.5 Hz, to 75.7 MPa and 87.4 MPa at 10 Hz, respectively. The mean storage modulus of anterior strut chordae decreased by 4.2–4.9 MPa over the frequency range following glutaraldehyde fixation.

**Table 2:** Viscoelastic properties of normal chordae under sinusoidally varying load across a range of frequencies

Frequency (Hz)	Anterior strut				Anterior basal				Posterior basal			
	Storage modulus (MPa)^b^		Loss modulus (MPa)		Storage modulus (MPa)^a^		Loss modulus (MPa)		Storage modulus (MPa)^a^		Loss modulus (MPa)	
	Mean	SD	Mean	SD	Mean	SD	Mean	SD	Mean	SD	Mean	SD
0.50	52.3	19.9	2.60	1.24	108	44.5	5.78	3.96	109	31.5	5.47	2.95
1.00	53.6	20.7	2.77	1.33	110	45.5	6.04	4.14	111	32.7	5.70	3.07
1.20	54.2	20.8	2.79	1.33	111	46.1	6.04	4.14	112	33.0	5.67	3.06
3.50	56.2	22.2	2.88	1.39	115	47.8	6.14	4.25	116	34.9	5.70	3.09
5.00	57.0	22.7	2.84	1.36	116	48.6	6.03	4.16	117	35.4	5.63	3.07
7.00	57.5	22.9	2.97	1.43	117	48.9	6.25	4.31	118	36.2	5.77	3.13
10.0	58.4	23.6	2.95	1.42	118	49.9	6.10	4.20	119	37.0	5.68	3.06

*n *= 8

*SD* standard deviation

The letters ^a, b^ are used to identify significant differences for storage modulus. If two chordal types do not share a letter, they are significantly different (*p* < 0.05) across all frequencies tested; note no significant difference was detected for loss modulus across chordal categories (*p* > 0.05)

**Table 3:** Viscoelastic properties of chordae treated with glutaraldehyde under a sinusoidally varying load across a range of frequencies

Frequency (Hz)	Anterior strut				Anterior basal				Posterior basal			
	Storage modulus (MPa)		Loss modulus (MPa)		Storage modulus (MPa)		Loss modulus (MPa)		Storage modulus (MPa)		Loss modulus (MPa)	
	Mean	SD	Mean	SD	Mean	SD	Mean	SD	Mean	SD	Mean	SD
0.50	47.9	13.3	2.31	1.43	67.3	20.9	3.63	2.47	77.7	33.3	4.29	3.33
1.00	49.3	13.8	2.54	1.55	69.0	21.4	3.85	2.58	79.5	33.8	4.50	3.48
1.20	49.9	14.0	2.53	1.55	70.2	21.7	3.89	2.58	80.6	34.3	4.55	3.52
3.50	51.7	14.4	2.73	1.67	72.8	22.4	3.99	2.66	83.8	35.4	4.70	3.62
5.00	52.2	14.6	2.71	1.66	73.8	22.7	3.95	2.66	85.0	35.8	4.70	3.63
7.00	52.8	14.9	2.93	1.78	74.8	23.0	4.08	2.72	86.5	36.5	4.94	3.79
10.0	53.5	15.0	2.88	1.76	75.7	23.3	4.07	2.73	87.4	36.9	4.87	3.74

*n *= 8

*SD* standard deviation

**Fig. 3:**
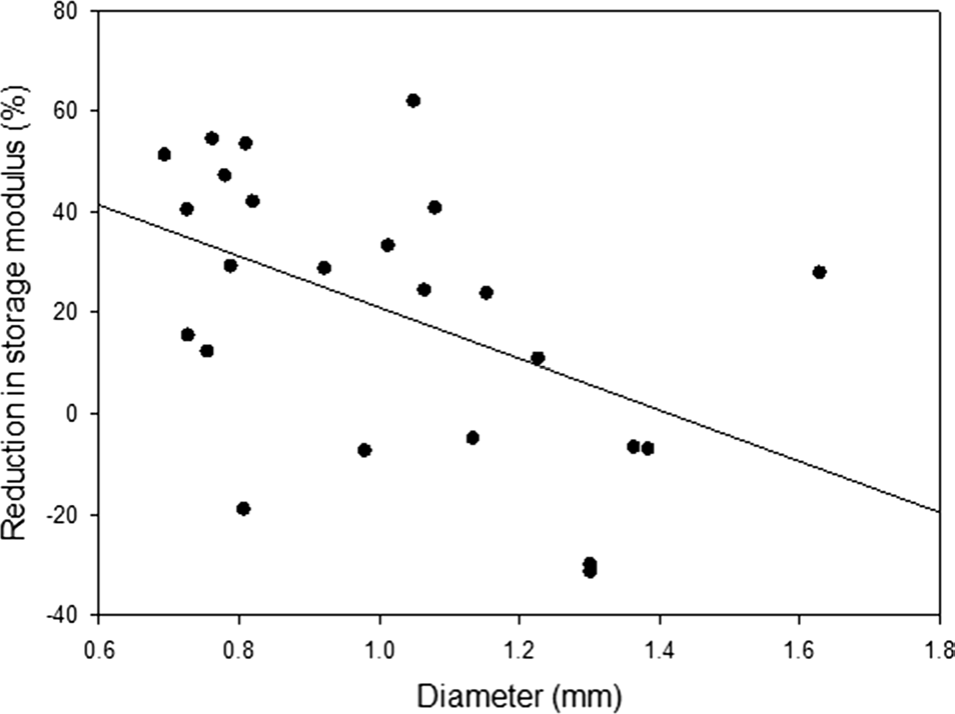
Regression of chordal diameter against reduction in storage modulus (percentage,  %) for 24 specimens at 1 Hz. Linear regression analysis determined the relationship to be significant (p = 0.018) with an R2 value of 0.2293. Note, any percentage reduction below 0% refers to an increase in storage modulus

Fixation with glutaraldehyde resulted in a significant (*p* < 0.05) decrease in the mean storage modulus of anterior basal chordae of 40.5–42.3 MPa over the frequency range. A mean storage modulus decrease of 30.8–31.9 MPa over the frequency range was present because of glutaraldehyde fixation for posterior basal chordae. The influence of chordal diameter on the decrease in storage modulus was evaluated at 1 Hz (Fig. 3). As chordal diameter increases, the change in storage modulus ($\Delta {E^{\prime}}$) due to fixation decreases.

Prior to fixation there were significant (*p *< 0.05) differences in the storage modulus of anterior strut chordae and both anterior and posterior basal chordae. The storage modulus of basal chordae was 56.2 MPa at 0.5 Hz, and 60.9 MPa at 10 Hz which was greater than that of anterior strut chordae (Table 2). Following fixation, there were no statistically significant differences between each of the chordal types (*p* > 0.05).

### Loss modulus

Regression analysis for the loss modulus demonstrated that 10 chordae were frequency independent (*p* > 0.05) whilst the remaining 14 were frequency dependent (*p* < 0.05). From the 14 frequency dependent results, 12 increased with increasing frequency, whilst two decreased. The loss modulus is represented by Eq. 8 below, where *C* and *D* are coefficients (Table 1).

8 $$E'' = C\ln ( f) + D$$

**Fig. 4:**
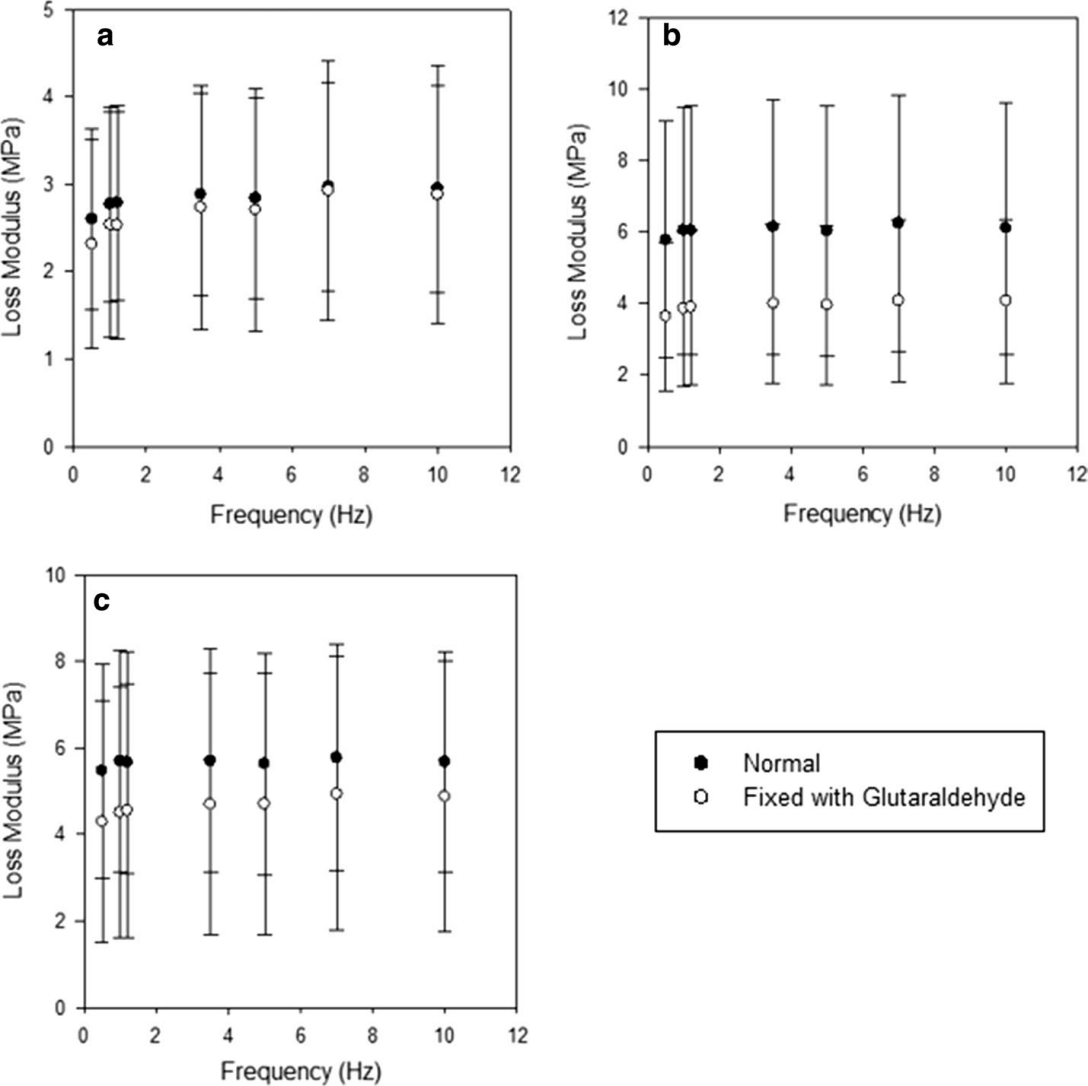
The effects of glutaraldehyde fixation on the loss modulus of **a** Anterior strut chordae, **b** Anterior basal chordae, and **c** Posterior basal chordae. Points represent the sample means with the 95% confidence intervals included. Means were calculated based on 8 samples from 4 different hearts for each chordal type. Black dots refer to unfixed chordae, while white dots represent glutaraldehyde treated chordae

The mean loss modulus ranged from: 2.60 MPa at 0.5 Hz, to 2.97 MPa at 7 Hz (Fig. 4a) for normal anterior strut chordae; 5.78 MPa at 0.5 Hz, to 6.25 MPa at 7 Hz (Fig. 4b) for normal anterior basal chordae; 5.47 MPa at 0.5 Hz, to 5.77 MPa at 7 Hz (Fig. 4c) for normal posterior basal chordae.

Fixation decreased the loss modulus of all chordae (Fig. 4) whilst affecting the frequency dependency. Following fixation, 22 specimens were frequency dependent (Eq. 8) while 2 remained frequency independent. The mean loss modulus of fixed anterior strut chordae ranged from 2.31 MPa at 0.5 Hz, to 2.93 MPa at 7 Hz. Following fixation, the mean loss modulus of anterior and posterior basal chordae ranged from 3.63 MPa and 4.29 MPa at 0.5 Hz, to 4.08 MPa and 4.94 MPa at 7 Hz (Fig. 4).

Fixation resulted in a significant (*p* < 0.05) decrease in the mean loss modulus of 2.15–2.17 MPa over the frequency range for anterior basal chordae. Fixation also resulted in a significant (*p *< 0.05) decrease in the mean loss modulus for anterior strut chordae of 0.29 MPa at 0.5 Hz. The decrease in loss modulus following fixation for the remaining results was not significant (*p* > 0.05).

For unfixed chordae, the minimum difference in loss modulus ($\Delta {E^{''}}$) between chordal types was 3.18 MPa at 0.5 Hz and the maximum difference 3.28 MPa at 7 Hz. This was reduced to a minimum difference of 1.97 MPa at 1 Hz and a maximum difference of 2.02 MPa at 1.2 Hz following glutaraldehyde fixation. Although the difference in loss modulus was not significant (Tables 2 and 3) before or after glutaraldehyde fixation, it can be observed from Fig. 4 that the loss modulus for anterior strut chordae was lower over the frequency range than that of other basal chordae.

## Discussion

### Overview

The effects of glutaraldehyde fixation on the viscoelastic properties of mitral valve chordae tendineae have been characterised. Both the storage and loss modulus decreased following glutaraldehyde fixation. The storage modulus of normal and fixed chordae were frequency dependent. Prior to fixation, the loss modulus of chordae varied across the range of specimens, showing a mix of frequency dependent and frequency independent results. Over a wider range of frequencies, it is possible that a correlation between loss modulus and frequency might be more evident. Following fixation, the overall trend showed chordae as frequency dependent, with the loss modulus increasing with frequency. The storage and loss modulus of basal chordae was larger than that of the anterior strut chordae before and after fixation. As diameter increased, the change in storage modulus due to fixation decreased.

### Effect of cross-linking on the dynamic viscoelasticity of chordae

The viscoelastic properties of mitral valve chordae have previously been characterised by their storage and loss moduli [11, 28]. Wilcox et al. [11] found that the storage modulus increased with frequency, whilst Lim et al. [28] found it to be frequency independent. The results from our current study are consistent with the findings that the storage modulus increases with frequency. However, the results are somewhat contradictory with respect to the loss modulus. Lim et al. [28] described the loss modulus to decrease with frequency, explaining that this would allow the valve to ensure complete closure at high heart rates. The results from our study are inconsistent with Wilcox et al. [11] and Lim et al. [28], such that the loss modulus of 12 specimens increased with increasing frequency. Alternatively, 10 specimens were frequency independent, consistent with Wilcox et al. [11], whilst the remaining 2 decreased with increasing frequency. Though reasons for this inconsistency are not obvious, the discrepancy obtained by Lim et al. [28] could be the result of human specimens being used as opposed to porcine specimens used by ourselves and Wilcox et al. [11].

For all chordae, the storage modulus was greater than that of the loss modulus across all frequencies, a trend observed in previous investigations [11, 28]. However, the magnitude of the results differs. Wilcox et al. [11] noted that small increases in load result in large increases in the storage modulus. The results from the investigation are within the range of 52.3–119 MPa for the storage modulus and 1.24–4.31 MPa for the loss modulus. The storage moduli are within the range identified in literature [11, 28] and align closely with the range identified by Lim et al. [28] between 20 and 140 MPa.

Glutaraldehyde fixation decreased the storage and loss modulus of chordae. Since knowledge of the effects of glutaraldehyde treatment on the dynamic properties of mitral valve chordae is limited, the following hypothesis is proposed. A link between the fibril crimp of collagen and their extensibility has been characterised [6]. It was shown that thicker chordae had a smaller crimp period than thinner chordae, and thus were more highly crimped. This has been hypothesised to characterise differences between the storage modulus of thick and thin chordae, whereby thicker chordae have a lower storage modulus [11]. Glutaraldehyde fixation is assumed to cause a chemical reaction between the aldehyde groups of glutaraldehyde and the ε-amine groups of lysine and hydroxylysine present in collagen [47, 48]. The result of this reaction is the formation of cross-links between the collagen fibres, which is thought to increase the fibril crimp, providing an explanation for the results (i.e. decrease in storage modulus). Further, since fixation is thought to increase the fibril crimp [23], we can extrapolate from these inferences to hypothesise that as thinner chordae become more predisposed to additional cross-linking, they experience a greater change in storage modulus; consistent with our current results. Thus, assuming fixation causes the chordae to become more crimped, we would expect the storage modulus to decrease due to fixation, this is reflected in the results for unfixed and fixed chordae.

Our results have shown that all three chordal types tested exhibited a decrease in storage modulus. However, the magnitude of the decrease varied. Although strut chordae are categorised as a type of basal chord, their diameter is larger, with strut diameter ranging from 0.7 to 1.1 mm as opposed to the diameter for other basal chordae ranging from 0.41 to 0.84 mm [11, 37]. Liao and Vesely [6] used a simple model to detail the effects of chordal diameter on possible linkages between fibrils. They concluded that the smaller the diameter, the greater number of fibril linkages that can occur. Consequently, the magnitude of fibril linkages that could occur within basal chordae would be larger than strut chordae. Thus, we hypothesise that increased fibril linkages would be expected to increase the storage moduli of chordae; the caveat being that if fibril linkages are increased via a chemical fixation process, then there may be a trade-off with changes in crimp (as described above).

The results from this study clearly show that the loss modulus of chordae decreased following fixation with glutaraldehyde. To explain these changes, we can consider the impact on the fibre matrix. It has previously been suggested that the loss modulus of chordae is related to the fibre matrix interactions [11], though this has not been investigated. Previously, we have only considered the impact of glutaraldehyde on the collagen molecules, however, since research into the effects of fixation of chordae is limited, evidence to support this is not currently conclusive. Since chordae have a complex structure made up of fibres (collagen; 65% dry weight) and elastin (9.7% dry weight) as well as proteoglycans (glycosaminoglycans; GAGs), the effects of fixation on more than just the collagen [8, 11, 49] should potentially be considered. Consequently, it is possible that these changes in the loss modulus because of glutaraldehyde fixation are due to changes occurring during potential fibre–GAGs interaction, not just the collagen molecules [11], though evidence to support this is currently limited.

### Clinical implications

Glutaraldehyde fixation alters the viscoelastic properties of chordae. Clinically, this would affect the functionality of a putative bioprosthetic replacement. Though marginal chordae were not considered during this investigation, it is likely that fixation would affect the tissues similarly. However, the crimp period of marginal chordae is larger than that of basal and strut chordae [6]. Consequently, fixation could cause marginal chordae to become more highly crimped. This is further supported by the hypothesis that chordae with smaller diameters are susceptible to larger amounts of fibril linkages. Thus, it is possible the reduction in stiffness of marginal chordae would exceed that of basal and strut chordae. This is of importance due to the function of marginal chordae in ensuring valve closure [11, 40, 50]. Though a decrease in stiffness for basal and strut chordae will be less critical, a decrease in marginal chordae stiffness could result in insufficient closure, consequently leading to valve failure [12].

Future studies considering the effect of fixation on chordal types would allow for complete characterisation of the effects of glutaraldehyde fixation on mitral valve chordae. Since alternative fixatives are under investigation, considering the effect of these alternatives on the dynamic properties would be of importance for bioprosthetic functionality [17].

## Conclusion

Fixation with glutaraldehyde decreased the magnitude of storage and loss properties of mitral valve basal chordae tendineae, which characterise its viscoelasticity. The storage and loss moduli of chordae decreased following glutaraldehyde fixation for all basal chordal types. The storage modulus of chordae before and after fixation remained frequency dependent. However, following fixation the frequency-dependency of the loss moduli of chordae was altered to a, typically, increased loss modulus with frequency.
